# The Effect of the Team Members Teaching Design vs. Regular Lectures method on the Self-efficacy of the Multiple sclerosis Patients in Iran. Randomised Controlled Trial

**DOI:** 10.17533/udea.iee.v42n2e13

**Published:** 2024-07-09

**Authors:** Ali Dehghani, Fariba Fakhravari, Mohsen Hojat

**Affiliations:** 1 Ph.D. Associate Professor, Department of Community Health Nursing. Email: ali.dehghani2000@gmail.com. Corresponding author. Jahrom University Department of Community Health Nursing Iran ali.dehghani2000@gmail.com; 2 M.Sc. Department of Nursing. Email: fariba.fakhravari@yahoo.com Jahrom University Department of Nursing Iran fariba.fakhravari@yahoo.com; 3 Ph.D. Assistant professor, Department of Nursing. Email: mohsenhojat@gmail.com Jahrom University Department of Nursing Iran mohsenhojat@gmail.com; 4 School of Nursing, Jahrom University of Medical Sciences, Jahrom, Iran. Jahrom University School of Nursing Jahrom University of Medical Sciences Jahrom Iran

**Keywords:** education, self-care, team members teaching design, reading, self-efficacy, multiple sclerosis, educación, autocuidado, miembros del equipo enseñando diseño, lectura, autoeficacia, esclerosis múltiple., educação, autocuidado, membros da equipe ensinando design, leitura, autoeficácia, esclerose múltipla

## Abstract

**Objective.:**

This study was conducted with the aim of the effect of team members teaching design (TMTD) vs. regular Lectures method on the self-efficacy of the multiple sclerosis patients.

**Methods.:**

This research is a randomized controlled trial study. In this study, 48 multiple sclerosis persons of members of Jahrom MS Society participated. The persons were selected by simple random sampling and then divided into three groups of: TMTD (*n*=16), regular lecture method (*n*=16), and control (*n*=16), by random allocation method. In the intervention groups, six training sessions were held twice a week; control group did not receive education. Data was collected by the MS self-efficacy questionnaire of Rigby *et al*. in the before, immediately and one month after the intervention.

**Results.:**

Patients in three intervention and control groups were similar in terms of demographic variables. The results of the repeated measurement test before, immediately and one month after the intervention showed that the mean of the all dimensions of self-efficacy in two intervention groups had increased significantly (*p*<0.05). While these changes were not significant in the control group (p ≥ 0.05). Also, there was a significant difference in the mean of the all dimensions of self-efficacy between the intervention groups of TMTD and regular lectures.

**Conclusion.:**

Based on the findings, TMTD compared to regular lectures method had a more significant effect on improving the self-efficacy of multiple sclerosis patients. Therefore, it is recommended that nursing use this educational approach to increase patients' self-efficacy.

## Resumen

**Objetivo.:**

Determinar el efecto del diseño de la enseñanza colaborativa de los miembros del equipo (En inglés: Team Members Teaching Design -TMTD) frente al método de las clases regulares sobre la autoeficacia de los pacientes con esclerosis múltiple (EM).

**Métodos.:**

Ensayo controlado aleatorizado realizado con la participación de 48 personas con esclerosis múltiple afiliados a la Sociedad de Esclerosis Múltiple de Jahrom (Iran), que fueron seleccionados por muestreo aleatorio simple y luego asignados en forma randomizada en tres grupos, dos de intervención: TMTD (*n*=16) y método de clases regulares (*n*=16), y un grupo control (*n*=16). En los grupos de intervención se impartieron seis sesiones educativas (dos por semana); mientras que el grupo control no recibió educación. Se empleó el cuestionario de autoeficacia en EM de Rigby *et al.* en los momentos: antes, inmediatamente después de terminada la intervención y un mes de finalizada la misma.

**Resultados.:**

Los pacientes de los tres grupos de intervención y control eran similares en cuanto a variables demográficas. Los resultados de la prueba de medidas repetidas antes, inmediatamente y un mes después de la intervención mostraron que la media de todas las dimensiones de autoeficacia en los dos grupos de intervención había aumentado significativamente (p<0.05). Mientras que estos cambios no fueron significativos en el grupo de control (p ≥ 0.05). Además, hubo una diferencia significativa en la media de todas las dimensiones de autoeficacia entre los grupos de intervención de TMTD y clases regulare**s,** siendo mayor en TMTD**.**

**Conclusión.:**

El TMTD comparado con el método de clases regulares, tuvo un mejor efecto en el aumento de la autoeficacia de los pacientes con EM. Por lo tanto, se sugiere a enfermería utilizar este enfoque educativo para aumentar la autoeficacia de los pacientes.

## Resumo

**Objetivo.:**

Determinar o efeito do desenho de ensino colaborativo dos membros da equipe (em inglês: *Team Members Teaching Design* -TMTD) comparado ao método de aulas regulares na autoeficácia de pacientes com esclerose múltipla (EM).

**Métodos.:**

Ensaio controlado randomizado realizado com a participação de 48 pessoas com esclerose múltipla afiliadas à Sociedade de Esclerose Múltipla de Jahrom (Irã), que foram selecionadas por amostragem aleatória simples e depois distribuídas aleatoriamente em três grupos, dois grupos de intervenção: TMTD (*n* =16 ) e método de aula regular (*n*=16), e um grupo controle (*n*=16). Foram ministradas seis sessões educativas nos grupos de intervenção (duas por semana); enquanto o grupo de controle não recebeu educação. Foi utilizado o questionário de autoeficácia em SM de Rigby et al. nos momentos: antes, imediatamente após o término da intervenção e um mês após seu término.

**Resultados.:**

Os pacientes dos três grupos intervenção e controle foram semelhantes em termos de variáveis ​​demográficas. Os resultados do teste de medidas repetidas antes, imediatamente e um mês após a intervenção mostraram que a média de todas as dimensões da autoeficácia nos dois grupos de intervenção aumentou significativamente (*p*<0.05). Embora essas alterações não tenham sido significativas no grupo controle (*p* ≥ 0.05). Além disso, houve diferença significativa na média de todas as dimensões de autoeficácia entre os grupos de intervenção TMTD e aulas regulares, sendo maior no TMTD.

**Conclusão.:**

O TMTD comparado ao método de aula regular teve melhor efeito no aumento da autoeficácia dos pacientes com EM. Portanto, sugere-se que a enfermagem utilize essa abordagem educativa para aumentar a autoeficácia dos pacientes.

## Introduction

Multiple sclerosis (MS) is a chronic inflammatory and autoimmune disease of the central nervous system that affects a person's sensory and motor function.^1^ This disease is one of the most common neurological diseases in humans and the second leading cause of disability in young adults after trauma. The most common age of the disease is 20 - 40 years. In fact, when a person is in the productive stage of life and can be effective for himself and society, he suffers from disability caused by this disease.^2^ So that it is the third cause of disability in America.^3^

MS affects more than 2.8 million people worldwide. Approximately 500 000 people in the United States have MS, and 8000 new cases are diagnosed each year.^4^ The prevalence of MS in Iran has been reported from 5.3 to 89 people per 100 000 people. According to the study of Azimi *et al*.^5^ the prevalence of MS in Iranian women was estimated at 16.5 per 100 000 people and in Iranian men at 14.8 per 100 000 people. MS patients experience a wide range of physical symptoms such as fatigue, immobility, weakness, tremors, pain, spasms, visual and sexual disturbances and psychologically, they suffer from cognitive impairment, depression, anxiety, reduced social interaction and increased dependence on others.^6^ Complications in MS are high and the most common side effects include psychological symptoms such as depression and anxiety and physical problems such as high blood pressure, hyperlipidemia, and chronic lung diseases.^7^ The changes caused by the disease strongly affect the self-efficacy of these patients. Therefore, it is necessary to pay attention to the self-efficacy of MS patients, which directly affects daily performance, social interactions, professional status, and quality of life.^8^High self-efficacy increases the quality of life and general health and reduces pain, fatigue, depression and stress.^9^ Self-efficacy is a person's belief in her abilities to organize and implement a set of activities necessary to achieve a specific outcome.^10^ Wilski study showed that individual factors such as general self-efficacy and perception of treatment control are more correlated with self-management in MS patients than clinical variables such as severity, type and duration of the disease.^11^

Since MS patients experience a lower level of self-efficacy than other people due to physical limitations and mental pressures caused by the disease, therefore, the implementation of interventions that can be accompanied by the implementation of a self-management program in these patients will improve self-efficacy.^12^ One of the interventions that can be implemented in the field of empowering patients to achieve self-management by the healthcare system is educational interventions. According to Naeemi*et al.,*^13^ educational interventions can improve awareness and self-efficacy for pain control among MS patients. One of the educational methods used by nurses and other health care professions which improves the patient's health behavior is self-care education. Self-care education explains why and how people should take care of themselves. Therefore, it is very important to use new methods in self-care education in order to improve the self-efficacy of patients to achieve the desired health behavior and control the complications of the disease. It is important to choose the appropriate educational method for self-care education.^14^Regarding MS disease, most of the educational programs that are provided to the patients are individual and in the form of pamphlets, guidance booklet and questions and answers.^15^

Often, due to the large number of patients in the MS Societies and the small number of trained personnel, the quality of educational programs is not very favorable and is not responsive to the patients. Often, trainings are presented in the lecture method, which results in quick forgetting of material, fatigue of learners, lack of opportunity for questions and answers, and lack of motivation in patients. This is despite the fact that today it is an effective education that is accompanied by positive activities of the learner and leads to the acquisition of constructive experiences in the patient.^16^ One of the new educational approaches is the use of team and cooperative teaching methods. Team Members Teaching Design (TMTD) is one of the team-teaching methods. Two hypotheses form the basis of the TMTD; the first hypothesis is that each member of the team studies a different part of the subject. Second, each learner can teach her team members, so each member acts as both a teacher and a learner.^17^The TMTD in small groups can lead to the creation of an active learning environment for all learners. The National League for Nursing believes that if people are going to work with each other in a quality way, they should train with each other to understand their common goals. One of the methods of implementing cooperative training is to use the skills of working groups. The role of teamwork in nursing patients is very important. In the clinical environment, 70-80% of errors occurred due to human causes, which were associated with poor teamwork. Therefore, doing group work is one of the very important educational strategies in nursing education programs, which can create and strengthen the spirit of cooperation, acquire social skills, and improve the professional skills of nursing students and nurses.^17^ Regarding the comparison of lecture and collaborative teaching methods such as TMTD, different and sometimes contradictory results have been obtained in the studies.^18^ So that the results of Sharifzade study found the TMTD to be more effective than lecture,^19^ and on the contrary, the study of Payami Bousari *et al*.^20^ showed that lecture method more effective than TMTD. 

Therefore, considering that use of collaborative method has been repeatedly emphasized in studies^20^^,^^21^ and taking into account that different results have been obtained in the comparison between the lecture teaching method and TMTD and so far, the TMTD has not been used in the training of patients. Hence, this study was conducted with the aim of the effect of the TMTD vs. regular Lectures method on the self-efficacy of the multiple sclerosis patients.

## Methods

**Figure 1:**
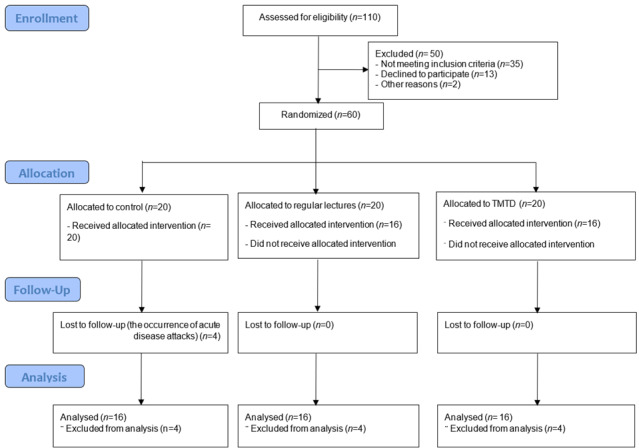
Flow diagram of the study

This research is a double-blind randomized controlled trial study with code IRCT20190127042506N1. The study was conducted in Jahrom MS Society in 2022. The inclusion criteria included definite diagnosis of MS, age 18-60 years, at least 6 weeks passed since the last relapse, self-care ability, and experience of MS for at least 2 years and willingness to participate in the study. Exclusion criteria included absence of more than 2 sessions during the intervention, occurrence of acute disease attacks during the intervention, and having heart, kidney, respiratory, digestive, and metabolic diseases. In order to select the patients to enter the study, first, among the patients who are members of the MS Society who met the criteria for entering the study, the samples were selected randomly, and then in the next step, using a computer, they were randomly allocated to two intervention (TMTD and regular lecture method) and control groups (Figure 1). The sample size was calculated based on Omidi *et al.*^22^ and considering α=0.05 and β=0.1, the number of 14 patients in each group was calculated. Considering the possibility of dropping samples during the study, 20 people were considered for each group. The formula for calculating the sample size is given below.




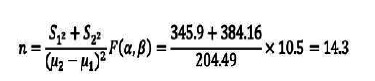




### Data collection tool

The tools used in this research were the questionnaire of demographic characteristics and the standard questionnaire of self-efficacy of MS patients. Demographic information questionnaire included age, sex, education, marriage, experience of disease, number of relapses and hospitalizations in the last year. MS self-efficacy questionnaire was developed by Rigby *et al*. ^23^in 2003. This scale includes 14 items and four dimensions of independence and activity (5 items), concerns and interests (4 items), personal control (3 items) and social efficiency (2 items). The range of scores varies from 14 - 84. Higher scores mean more self-efficacy. The scoring of this scale is done on a 5-point Likert scale from completely disagree (score 1) to completely agree (score 5). This scale has been translated and validated in Iran by Tanhaye Reshvanlo and Soleimanian.^24^ The number of items was reduced from 14 items to 11 items by removing 3 items due to the factor loading less than 0.3 and classified into 3 dimensions. Cronbach's alpha coefficient of the whole questionnaire is reported 0.90. Cronbach's alpha coefficients for three dimensions of independence and activity, personal control, concerns and interests were obtained 0.80, 0.78, and 0.72 respectively.

### Intervention

Before starting the study, demographic information and MS self-efficacy questionnaire were completed by patients in all three groups. Then the educational intervention was held for the intervention groups in six educational sessions and twice a week (On Sundays, the regular lecture method and on Tuesdays, TMTD). The training sessions lasted for six weeks. The training in the regular lecture group included the presentation of materials in the lecture method by the researcher. The steps of TMTD were implemented as follows: In the first session, the patients were divided into four teams of 5 people (due to the non-participation of 4 patients in the training sessions, the sessions continued in four groups of 4 people). The researcher prepared content about self-care training and divided them into equal volumes and distributed them to all patients a week before, and from the patients were requested to read the content. On the day of the training session, according to the previous division of the members, the first members of each of the four teams in one category, the second members of each team in one category, the third members of each team in another category, and the fourth members of each team in another category were placed. In fact, four groups of four people were formed, and each group had to present a single topic in their main teams. Then the patients were asked to discuss, exchange opinions and understand the contents for 10 minutes with all four people who have the same content in each of the categories. After the completion of the first stage, all members returned to their original teams and for 10-15 minutes, each of them presented their respective content in their teams.^18^At the end, the researcher summarized the contents and answered the patients' questions.

The content of the six training sessions in both the regular Lectures method and TMTD was as follows: The first session: Familiarizing patients with each other, talking about self-care and its importance in controlling disease complications. The second session: Familiarizing patients with the proper diet in MS and following a healthy diet and limiting certain foods. The third session: Familiarity with pain management methods and fatigue reduction and muscle relaxation methods. The fourth session: Familiarity with methods of improving urinary disorders in patients. The fifth session: Familiarity and coping with stress, anxiety and depression and the sixth session: Familiarity with exercise in MS disease (Table 1). After the completion of the educational sessions, the patients in the intervention groups were followed up for one month, and during this period, the researcher attended the MS Society and answered the patients' questions by phone and in person. Immediately and one month after the intervention, the self-efficacy questionnaire was completed by the patients of all three groups. Patients in the control group did not receive education.

### Statistical analysis

Data analysis was done using SPSS 22. Homogenization of the samples in terms of demographic variables in the intervention and control groups was done using chi-square and independent t-test. Normality of the data was done using the Kolmogorov-Smirnov test. In order to compare the mean in the three intervention (lecture and TMTD) and control groups, the multivariable Analyze of covariance test (MANOVA) was used. For the compare the mean in the pre and post-intervention, the paired t-test was used. Also, in order to investigate the changes trend before, immediately and one month after the intervention in the three intervention and control groups, the repeated measures analysis was used. A significance level of 0.5 was considered.

### Ethical Issues

This study was approved by the ethics committee of Jahrom University of Medical Sciences with ethics code IR.JUMS.REC.1397.152. A written informed consent form was completed by the patients. The research samples were assured about the anonymity of the questionnaire, privacy and confidentiality, and voluntary participation in the research. Also, the purpose of the study was explained to the patients in the intervention groups.

**Table 1:** Description of educational programs in the two intervention groups (TMTD and regular lecture methods)

Session	Location	Content	Duration of session
1	Conference room of MS Society	Familiarizing patients with each other, talking about self-care and its importance in controlling disease complications.	One hour
2	Conference room of MS Society	Familiarizing patients with the proper diet in MS and following a healthy diet and limiting certain foods.	One hour
3	Conference room of MS Society	Familiarity with pain management methods and fatigue reduction and muscle relaxation methods.	One hour
4	Conference room of MS Society	Familiarity with methods of improving urinary disorders in patients.	One hour
5	Conference room of MS Society	Familiarity and coping with stress, anxiety and depression.	One hour
6	Conference room of MS Society	Familiarity with exercise in MS disease.	One hour

## Results

12 patients (total of 60 patients) were excluded from the study due to reasons such as absence in the training sessions and occurrence of acute disease attacks. Therefore, the findings were analyzed with 16 patients in each group (48 patients in total). The analysis of the findings showed that the patients in the intervention and control groups were identical in terms of demographic variables. The average age of the patients in the TMTD group was 39 ± 12.143, the regular lecture group was 33.12 ± 10.38, and the control group was 38.62 ± 7.46, which had no significant difference. Other demographic characteristics are given in Table 2.

**Table 2:** Demographic characteristics of MS patients in intervention and control groups

*p*-value	Control			Lecture		TMTD		Group		Variable
	%	*n*	%	*n*	%	*n*		
0.881	81.3	13	75	12	81.3	13	Female	Gender
	18.8	3	25	4	18.8	3	Male	
0.889	25	4	18.8	3	25	4	Single	Marriage
	75	12	81.3	13	75	12	Married	
0.102	25	4	25	4	43.8	7	Under diploma	Education level
	25	4	37.5	6	50	8	Diploma	
	50	8	37.5	6	6.3	1	Upper diploma	
0.521	56.3	9	68.8	11	81.3	13	Housewife	Job
	12.5	2	6.3	1	0	0	Employee	
	31.3	5	25	4	18.8	3	Freelance	
0.281	50	8	43.8	7	37.5	6	No recurrence	The frequency of disease recurrence in the past year
	18.8	3	31.3	5	37.5	6	One	
	31.3	5	18.8	3	6.3	1	Two	
	0	0	6.3	1	18.8	3	More than two	
0.162	56.3	9	37.5	6	50	8	0	Number of hospitalizations in the past year
	18.8	3	31.3	5	6.3	1	1	
	18.8	3	31.3	5	18.8	3	2	
	6.3	1	0	0	25	4	≥3	
	Mean ± SD	Mean ± SD	Mean ± SD		
0.216	38.62 ± 7.46	33.12 ± 10.38	39 ± 12.143		Age (years)
0.554	8.68 ± 5.95	8 ± 7.54	9.25 ± 6.64		The experience of disease

The results of the Kolmogorov Smirnov test showed that the data in the intervention and control groups had a normal distribution before, immediately and one month after the intervention. Therefore, parametric tests of multivariable analyze of covariance, paired t-test and repeated measurement test were used to analyze the data. The results of the repeated measurements showed that the mean of the self-efficacy and its dimensions in both TMTD and regular lectures methods immediately and one month after the intervention had a significant increase compared to before the intervention, while no such change was observed in the control group (Table 3).

**Table 3:** Comparison of mean and standard deviation of scores of self-efficacy dimensions in intervention and control groups in three-time stages before, immediately and one month after the intervention

*p*-value	Control		Lecture		TMTD		Group	Self - efficacy dimensions
	SD	Mean	SD	Mean	SD	Mean		
0.08	2.79	12.75	4.61	13.43	2.89	13.12	Before intervention	Independence and activity
0.04	2.70	12.5	3.64	14.68	2.01	15.93	Immediately after the intervention	
0.001	2.80	12.37	3.28	15	1.43	16.25	One month after the intervention	
-	0.12	0.02	0.001	*p*-value^**^	
0.1	1.85	9.68	2.36	10.87	2.59	10.93	Before intervention	Personal control
0.02	1.96	9.12	2.12	11.43	2.09	12.43	Immediately after the intervention	
0.002	1.40	8.87	1.62	11.87	0.83	13.18	One month after the intervention	
-	0.08	0.002	0.01	*p*-value^**^	
0.12	3.68	13.62	3.89	14	3.84	13	Before intervention	Concerns and interests
0.001	3.66	13.37	3.27	15.06	2.18	16.37	Immediately after the intervention	
0.002	3.68	13.37	3.40	15.43	1.86	16.43	One month after the intervention	
-	0.11	0.02	0.002	*p*-value^**^	
0.06	6.87	36.06	10.37	38.31	6.77	37.06	Before intervention	Overall self-efficacy score
0.01	6.19	35	8.35	41.18	4.10	44.75	Immediately after the intervention	
0.002	5.71	34.62	7.34	42.3	2.68	45.87	One month after the intervention	
-	0.102	0.002	0.001	*p*-value^**^	

* Multivariable Analyze of covariance, ^**^Repeated measures analysis

A comparison of the changes in the intervention and control groups in the self-efficacy variable showed that there is a significant difference between the change trend of the control group and TMTD. There is a significant difference between the change trend of the control group and the lecture group. Also, there is a significant difference between the change trend of lecture groups and TMTD. Examining the difference between the mean scores before and after the intervention of intervention groups, TMTD and lectures with the scores of the control group, indicates the effectiveness of self-care training in both teaching methods of TMTD and lectures on patients' self-efficacy. Considering that there is a significant difference between the intervention groups of TMTD and lectures; therefore, the superiority of self-care training is determined with the TMTD over the lecture method (Table 4).

**Table 4:** Comparison of changes in self-efficacy variable among groups

*p*-value	Standard error	Average difference	Compared groups	Variables
0.001	1.40	-10.275	Control-TMTD	Self - efficacy
0.001	1.44	-5.621	Control - Lecture	
0.002	1.44	4.65	Lecture - TMTD

## Discussion

In this research, a comparative study of the effect of self-care training based on the TMTD and lectures on the self-efficacy of MS patients was done. The trend of changes in the mean of self-efficacy before, immediately and one month after the intervention showed that the mean of self-efficacy in the TMTD and lectures were significant in a positive direction. The significance of the trend of changes in the TMTD and lectures indicates the continuation of self-care training during the period of one month and it shows that the effects of training have not disappeared with the passage of time, but with the follow-up and implementation of training in the activities and daily life of the patients, self-efficacy has been improved within a month after the completion of the training sessions. The studies of Maslakpak and Raiesi,^25^ Daniali*et al*.^26^ Boosman*et al*.^27^ and Jongen *et al.*^28^ confirm the findings of the present study.

The results of Maslakpak and Raiesi study showed that the implementation of self-management program along with regular follow-up has increased self-efficacy in MS patients.^25^ Part of the self-management program used in this study, such as diet, stress and anxiety control, and physical activity, is consistent with the educational programs of the present study. The results of Jongen *et al*. ^28^showed that six months after a wellness program, MS patients with a relapse or low disability may experience improved self-efficacy and higher health-related quality of life. Therefore, with the patient's participation in self-care and supporting the patient in the implementation of health plans in the follow-ups carried out by the nurse and other health professionals, the patient can be helped to achieve independence and personal control. 

Also, the findings of this study showed that the TMTD had a more significant effect on the self-efficacy of MS patients compared to the lecture method. The results of the study by Borzou*et al.*^29^ about the comparison of individual and peer training on the quality of life of patients with heart failure showed that both training methods lead to an increase in the quality of life, but the effect of peer education is greater in the long term. The results of the study by Dehghani also showed that the lecture method and peer education both lead to the improvement of health literacy in MS patients. However, peer education compared to lectures has a more significant effect on the health literacy of MS patients,^30^ which is in line with the findings of the present study. In both methods of peer education and TMTD, there are common aspects such as patient participation in learning, provision of education by patients themselves to group members. In the study of Borzou*et al.*^29^ the results regarding the comparison of peer education with the face-to-face individual education showed that in individual education such as lecture method, the patient has a passive role in the learning process. In this regard, the results of Sharifzade study showed that the TMTD can effectively increase learning compared to the usual lecture method which is consistent with the findings of the present study.^19^

The results of a study conducted by Hassanzadeh *et al.*^31^showed that the use of cooperative methods is more effective than passive and traditional teaching methods such as lectures. The results of Rahimian *et al.*^32^Also showed that two months after the educational intervention, the mean of self-efficacy and its areas in the peer education group was higher than the lecture group. Considering the similarity of the peer education method with TMTD and also the chronicity of the disease of the participants in Rahimian *et al.* ^32^and the current research, perhaps the participation of patients in the method of peer education and TMTD compared to lectures has led to improved self-efficacy and self-care in patients.

According to the findings of the study, it is possible to familiarize nurses with the self-care training program in a team form by using methods such as holding a workshop and use team training methods in the subject of patient education, especially chronic diseases. On the other hand, the main goal in clinical nursing is to provide services to patients and help them improve, and education plays a significant role in this field. Therefore, it is possible to use the team training approach that leads to simple and reliable learning and increase the motivation and improvement of health behaviors of patients, as a valuable tool to improve, treat and control the physical and mental complications of patients in medical centers. Also, due to the shortage of nursing staff, the lack of time of nurses to provide care, patients' acceptance of learning through team teaching methods, and entrusting the process of education to patients with chronic diseases, it is possible to help them learn better by teaching team methods.^18^^,^^33^

The above studies showed that the educational programs proposed by the TMTD and cooperative education can more effectively encourage people to selection of appropriate health behaviors. ^18^^,^^33^^,^^34^ Also, these findings can be due to the fact that in the TMTD, patients belong to the same social group and people believe that they are of similar ability, so it can have a more important effect on learning. Another reason for the findings of the present study could be the different teaching methods in the two intervention groups. For example, in the TMTD, there are more group discussions, question and answer, and interpersonal interactions. While in the other intervention group, only the lecture method was used. Therefore, the learning is higher in patient-centered educational methods such as the TMTD compared to lectures, which is one of the advantages of active and patient-centered educational methods compared to traditional teaching methods such as lectures.^35^The drop in the number of samples present in the intervention and control groups, short-term follow-up after the intervention, differences in the personality characteristics and culture of the patients in accepting educational content are among the limitations of the present study.

Conclusion. The results of this study showed that the TMTD can increase self-efficacy among MS patients. Also, the findings of this study showed that the TMTD has a more significant role in improving the self-efficacy of MS patients compared to lectures. Therefore, the TMTD can be used as a beneficial educational-supportive method for MS patients.
